# Electrospun Fiber-Coated Human Amniotic Membrane: A Potential Angioinductive Scaffold for Ischemic Tissue Repair

**DOI:** 10.3390/ijms23031743

**Published:** 2022-02-03

**Authors:** Hanis Nazihah Hasmad, Ruszymah Bt Hj Idrus, Nadiah Sulaiman, Yogeswaran Lokanathan

**Affiliations:** Centre for Tissue Engineering and Regenerative Medicine, National University of Malaysia, Kuala Lumpur 56000, Malaysia; hanisnazihah@gmail.com (H.N.H.); ruszyidrus@gmail.com (R.B.H.I.); nadiahsulaiman@ukm.edu.my (N.S.)

**Keywords:** angiogenesis, electrospun fiber, human amniotic membrane, skeletal myoblast, ischemia

## Abstract

Cardiac patch implantation helps maximize the paracrine function of grafted cells and serves as a reservoir of soluble proangiogenic factors required for the neovascularization of infarcted hearts. We have previously fabricated a cardiac patch, EF-HAM, composed of a human amniotic membrane (HAM) coated with aligned PLGA electrospun fibers (EF). In this study, we aimed to evaluate the biocompatibility and angiogenic effects of EF-HAM scaffolds with varying fiber thicknesses on the paracrine behavior of skeletal muscle cells (SkM). Conditioned media (CM) obtained from SkM-seeded HAM and EF-HAM scaffolds were subjected to multiplex analysis of angiogenic factors and tested on HUVECs for endothelial cell viability, migration, and tube formation analyses. All three different groups of EF-HAM scaffolds demonstrated excellent biocompatibility with SkM. CM derived from SkM-seeded EF-HAM 7 min scaffolds contained significantly elevated levels of proangiogenic factors, including angiopoietin-1, IL-8, and VEGF-C compared to plain CM, which was obtained from SkM cultured on the plain surface. CM obtained from all SkM-seeded EF-HAM scaffolds significantly increased the viability of HUVECs compared to plain CM after five days of culture. However, only EF-HAM 7 min CM induced a higher migration capacity in HUVECs and formed a longer and more elaborate capillary-like network on Matrigel compared with plain CM. Surface roughness and wettability of EF-HAM 7 min scaffolds might have influenced the proportion of skeletal myoblasts and fibroblasts growing on the scaffolds and subsequently potentiated the angiogenic paracrine function of SkM. This study demonstrated the angioinductive properties of EF-HAM composite scaffold and its potential applications in the repair and regeneration of ischemic tissues.

## 1. Introduction

Skeletal myoblasts are an attractive cell source for cellular cardiomyoplasty because of their autologous availability, high proliferative properties, and high tolerance to ischemia [1]. The transplantation of skeletal myoblasts has demonstrated preclinical and clinical therapeutic benefits in restoring cardiac function after myocardial infarction (MI) [2,3,4]. An increase in ejection fraction, a higher degree of angiogenesis at the infarct site, and reverse ventricular remodeling have been observed in infarcted hearts following myoblast transplantation [2,5,6]. It has been suggested that the improvement in cardiac function observed following myoblast transplantation might be exerted through paracrine properties, rather than the replacement of damaged cardiac muscle with new contractile tissue [1].

Improving the survival of grafted cells helps to maximize the therapeutic paracrine effects of myoblast transplantation. Several strategies have been used to enhance the survival and engraftment efficiency of skeletal myoblasts in infarcted hearts. These strategies include transplantation of myoblast cell sheets or tissue-engineered scaffolds, genetic modification of skeletal myoblasts, and preconditioning of skeletal myoblasts prior to transplantation [7,8,9,10]. As previously reported, intramyocardial myoblast injection may trigger arrhythmic events in patients owing to the failure of skeletal myoblasts to electromechanically couple to the host myocardium, as they remain functionally isolated [11]. The use of myoblast cell sheets or tissue-engineered scaffolds may alleviate the risk of arrhythmia while facilitating cardiac functional recovery through consistent delivery of angiogenic, anti-apoptotic, and antifibrotic cytokines for a longer period of time.

Angiogenesis is indispensable for the recovery of ischemic tissue and is regarded as an effective therapeutic target for cardiac repair and regeneration. It has been reported that vessel ingrowth precedes cardiomyocyte migration during cardiac regeneration, and increased angiogenesis contributes to cardiomyocyte survival and suppresses ventricular remodeling in infarcted hearts [12,13]. Knowing that myoblast-secreted factors could contribute to the functional improvement of infarcted myocardium, cardiac patch seeded with skeletal muscle cells (SkM) could serve as an ideal reservoir of soluble proangiogenic factors for ischemic tissue repair. Therefore, understanding how the interaction between cardiac patch and SkM affects the secretion of proangiogenic factors is crucial for producing tissue-engineered cardiac patch with enhanced angiogenic potential.

In our previous study, we fabricated a composite scaffold, EF-HAM, comprising a human amniotic membrane (HAM) and electrospun PLGA fibers (EF) for aligned tissue engineering applications [14]. In addition to having good mechanical strength and elasticity, EF-HAM scaffolds also helped to maintain cell viability and have been shown to guide the orientation and migration of SkM owing to their aligned topography. We also demonstrated that electrospun fiber thickness influenced the alignment and diameter of fibers, and tensile properties of scaffolds. In the current study, we aimed to investigate the proangiogenic potential of SkM-seeded EF-HAM scaffolds for the future treatment of ischemic tissue by studying their angiogenic paracrine effects in vitro. For this purpose, we collected conditioned media (CM) from SkM cultured on HAM and EF-HAM scaffolds with varying fiber thicknesses and subjected them to multiplex paracrine factor analysis and functional assays using human umbilical vein endothelial cells (HUVECs) to evaluate the angiogenic stimulation of endothelial cell viability, migration, and tube formation.

## 2. Results

### 2.1. Physical Characteristics of EF-HAM Scaffolds

**Figure 1:**
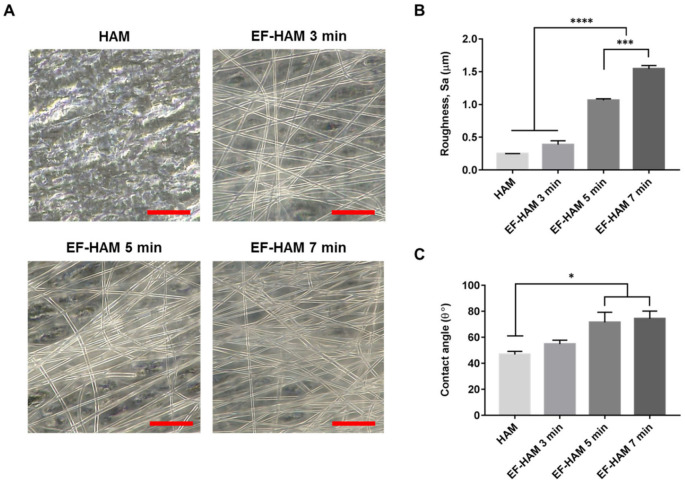
The gross appearance and characteristics of HAM and EF-HAM scaffolds. (**A**) PLGA fiber morphology on HAM with varying fiber densities, (**B**) measurements of surface roughness, and (**C**) water contact angle of the scaffolds. The values are represented as mean ± SEM (* *p* < 0.05, *** *p* < 0.001, or **** *p* < 0.0001; One-way ANOVA). Scale bar: 20 µm.

The fabrication of PLGA fibers on decellularized HAM with increasing deposition time produced EF-HAM scaffolds with thicker and denser fiber layers, as illustrated in Figure 1A. The increased deposition of PLGA fibers on HAM also resulted in higher surface roughness and water contact angle of the EF-HAM scaffolds. As shown in Figure 1B,C, EF-HAM 5 min and EF-HAM 7 min scaffolds had significantly rougher and less hydrophilic surfaces compared with HAM owing to the presence of thick PLGA fiber coating.

### 2.2. In Vitro Biocompatibility of EF-HAM Scaffolds

**Figure 2:**
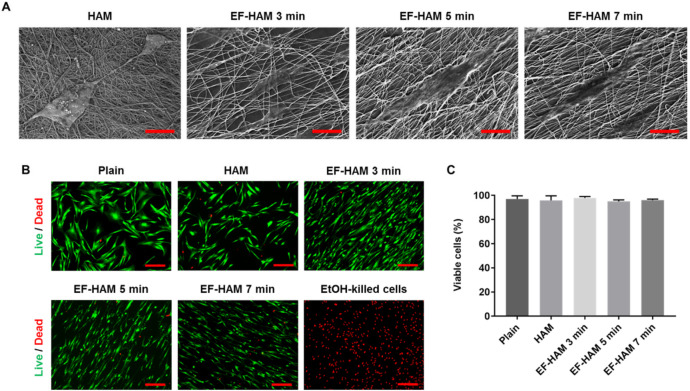
In vitro biocompatibility of HAM and EF-HAM scaffolds with skeletal muscle cells (SkM). (**A**) Electron micrographs showing good adhesion of SkM on HAM and EF-HAM scaffolds. (**B**) Live and dead assay images of SkM stained with Calcein AM and EthD-1 and (**C**) the percentage of viable cells attached to the scaffolds. The values are represented as mean ± SEM (One-way ANOVA). Scale bar: 40 µm, except for HAM (2 µm) (in Figure 2A); 200 µm (in Figure 2B).

Examination with FESEM revealed that SkM adhered to and spread well on the surface of HAM and all EF-HAM scaffolds (Figure 2A). Cells growing on EF-HAM scaffolds were attached to PLGA fibers and elongated along the alignment of the fibers, whereas cells growing on HAM were attached to the exposed extracellular matrix (ECM) and repopulated the membrane in a random orientation. Live and dead cell analysis confirmed that HAM and all EF-HAM scaffolds were biocompatible and did not exhibit cytotoxic effects against SkM, as the percentage of viable cells in each group was above 95% (Figure 2B,C).

### 2.3. CM Derived from SkM-Seeded EF-HAM 7 Min Scaffolds Contained Higher Levels of Angiopoietin-1, IL-8, and VEGF-C

**Figure 3:**
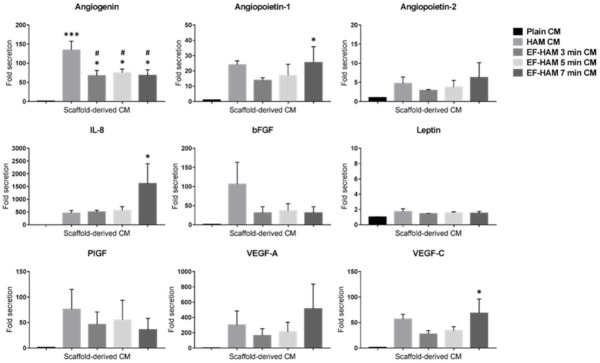
Detection of multiple proangiogenic factors secreted by SkM-seeded HAM and EF-HAM scaffolds. Conditioned medium (CM) from each experimental condition was collected in serum-free culture medium for 72 h before the proangiogenic factors were measured via multiplex analysis. The values are represented as mean ± SEM (* *p* < 0.05 or *** *p* < 0.001, compared with plain CM; # *p* < 0.05, compared with HAM CM; One-way ANOVA).

To establish the angiogenic paracrine potential SkM-seeded HAM and EF-HAM scaffolds, CM from each scaffold was collected and subjected to multiplex analysis of proangiogenic factors. Investigation of the angiogenic factors content secreted by SkM-seeded HAM and EF-HAM scaffolds revealed significantly elevated levels of several proangiogenic factors in the HAM and EF-HAM CM compared with those secreted by SkM cultured on the plain surface (plain CM) (Figure 3). The secretion level of angiogenin increased 134.5-fold in HAM CM and from 67.4-fold to 75.6-fold in different EF-HAM CM groups compared with that in plain CM. Angiogenin secretion was significantly higher in the HAM CM group than in all the EF-HAM CM groups. Notably, three specific proangiogenic factors, angiopoietin-1, IL-8, and VEGF-C, were found to be significantly elevated only in EF-HAM 7 min CM compared with plain CM. The fold-secretions of these factors against those in plain CM were 25.4-, 1611.3- and 68.5-fold, respectively. Other angiogenic factors, including angiopoietin-2, bFGF, leptin, PlGF, and VEGF-A, were present in CM derived from SkM-seeded HAM and EF-HAM scaffolds at various degrees of secretion, but the secreted levels were not significantly different when compared with plain CM.

### 2.4. CM Derived from SkM-Seeded EF-HAM Scaffolds Enhanced Endothelial Cell Viability

**Figure 4:**
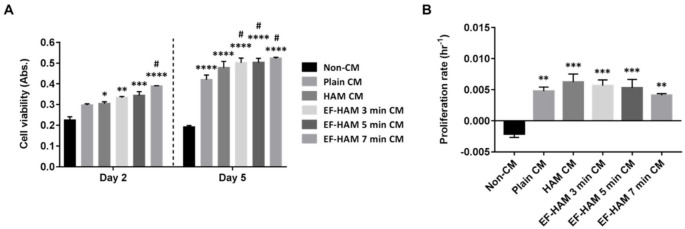
HUVECs grown in different EF-HAM CM showed higher viability than those grown in plain CM. (**A**) HUVEC viability measured using the PrestoBlue assay on the 2nd and 5th days of culture. (**B**) The proliferation rate of HUVECs was enhanced in the presence of CM. Two-way and one-way ANOVA were performed as shown in Figure 4A,B, respectively, as statistical tests for means and the values are represented as mean ± SEM (* *p* < 0.05, ** *p* < 0.01, *** *p* < 0.001, or **** *p* < 0.0001, compared with non-CM control; # *p* < 0.05, compared with plain CM).

Various coordinated events involving endothelial cell functions are responsible for angiogenesis, including matrix degradation, cell migration, proliferation, and morphogenesis. The angiogenic potential of CM from SkM-seeded HAM and EF-HAM scaffolds was studied by evaluating its influence on the viability of endothelial cells. As shown in Figure 4A, HUVECs subjected to HAM CM and all three different EF-HAM CM had significantly higher cell viability than those in the non-CM control group on the 2nd day of culture. On the 5th day, all CM-treated HUVECs maintained a significantly higher cell viability than non-CM-treated cells. Meanwhile, EF-HAM 7 min CM significantly increased HUVEC viability compared with plain CM as early as the 2nd day, followed by EF-HAM 3 min CM and EF-HAM 5 min CM by the 5th day. As shown in Figure 4B, HUVECs supplemented with CM either from SkM cultured on the plain surface or SkM-seeded HAM and EF-HAM scaffolds all showed increased proliferation compared with those in the non-CM control group, whereby proliferation was negatively influenced by reduced cell viability. Neither HAM CM nor EF-HAM CM induced a higher proliferation rate in HUVECs than plain CM.

### 2.5. CM Derived from SkM-Seeded EF-HAM 7 Min Scaffolds Induced Higher Migration Capacity in HUVECs

**Figure 5:**
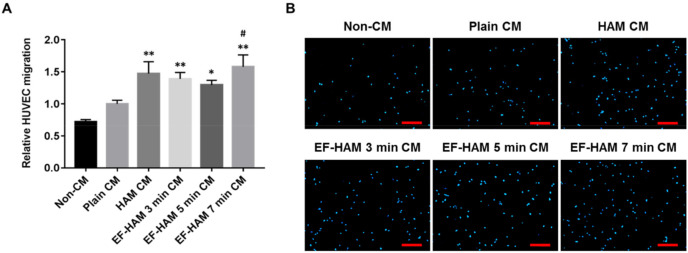
EF-HAM 7 min CM induced a higher transwell migration capacity in HUVECs than plain CM. (**A**) Relative HUVEC migration when co-cultured with SkM-seeded HAM and EF-HAM CM compared with plain CM. (**B**) Representative images of migrated HUVECs, nuclear-stained with DAPI, at the bottom of transwell inserts. The values are represented as mean ± SEM (* *p* < 0.05 or ** *p* < 0.01, compared with non-CM control; # *p* < 0.05, compared with plain CM; One-way ANOVA). Scale bar: 200 µm.

A transwell migration assay was conducted to determine whether angiogenic paracrine factors secreted by SkM-seeded HAM and EF-HAM scaffolds could exert a chemotactic response in HUVECs. As shown in Figure 5A,B, CM secreted from HAM and all EF-HAM tissue constructs significantly enhanced the migration capacity of HUVECs relative to that of their non-CM control. Notably, only EF-HAM 7 min CM managed to elicit a higher migratory response in HUVECs at almost 60% greater capacity than plain CM.

### 2.6. EF-HAM 7 Min CM Promoted Longer Tube Formation in HUVECs

**Figure 6:**
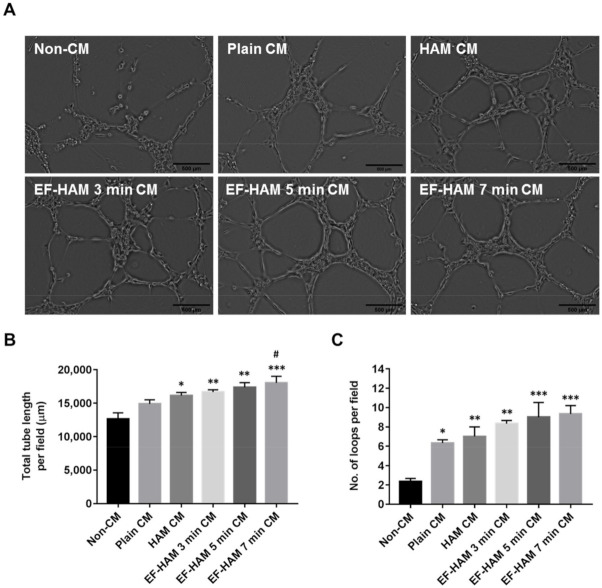
EF-HAM 7 min CM induced longer tube formation in HUVECs than plain CM. (**A**) Formation of capillary-like structures by HUVECs cultured in the presence of CM from different SkM-seeded scaffolds on Matrigel. (**B**) Total tube length and (**C**) number of loops per field were quantified to evaluate the proangiogenic effects of CM on endothelial tube formation. The values are represented as mean ± SEM (* *p* < 0.05, ** *p* < 0.01, or *** *p* < 0.001, compared with non-CM control; # *p* < 0.05, compared with plain CM; One-way ANOVA). Scale bar: 500 µm.

To further confirm the angiogenic potential of SkM-seeded HAM and EF-HAM scaffolds, the ability of CM obtained from each scaffold to stimulate tube formation in HUVECs was evaluated using a Matrigel-based assay. Images representing the reorganization of HUVECs into capillary-like structures were recorded and analyzed using an automated tube formation quantification software against several characteristic points and elements of the endothelial cell network (Figure 6A). CM derived from SkM-seeded HAM and all EF-HAM scaffolds significantly enhanced tube formation, as they increased both the total length of tubes and the number of loops compared with the non-CM control (Figure 6B,C). Plain CM also enhanced the number of loops compared with the non-CM control but did not affect the total length of the tubes formed. Notably, only CM secreted by the SkM-seeded EF-HAM 7 min scaffolds was found to significantly increase the total length of tubes formed when compared with plain CM.

### 2.7. SkM-Seeded EF-HAM 7 Min Scaffolds Had a Higher Proportion of Skeletal Fibroblasts

**Figure 7:**
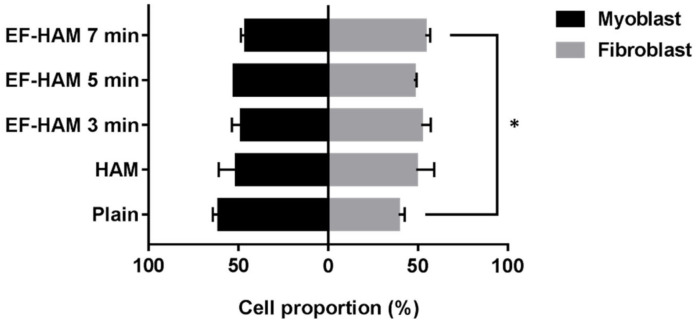
Proportion of skeletal myoblasts and fibroblasts growing on SkM-seeded HAM and EF-HAM scaffolds at the time of CM collection. The values are represented as mean ± SEM (* *p* < 0.05, compared with plain; One-way ANOVA).

Upon collection of CM from the SkM-seeded HAM and EF-HAM scaffolds, the proportion of skeletal myoblasts and fibroblasts on the scaffolds was evaluated to determine the effect of fibrous scaffold topography on the population of both cells. As illustrated in Figure 7, SkM cultured on EF-HAM 7 min scaffolds displayed a significantly higher proportion of skeletal fibroblasts (54%), whereas a greater proportion of skeletal myoblasts (61%) was observed in the SkM culture on plain surface.

## 3. Discussion

The use of biomaterials that closely mimic the microenvironment and anisotropic characteristics of the native myocardium improves the delivery, retention, and survival of therapeutic cells at the infarct site. These biomimetic materials can potentiate the secretion of soluble factors with anti-inflammatory, proangiogenic, and anti-apoptotic properties that would mediate cardiac tissue repair and regeneration. Hence, identifying the most compatible biomaterial is crucial for the development of functional cardiac patches.

Both human amniotic membrane (HAM) and electrospun nanofibers have been extensively used for wound healing and multiple tissue regeneration applications, but as separate scaffolds on their own. HAM has a long clinical history of being used as a wound dressing and as a substrate for ocular surface reconstruction owing to its strong elastic nature and favorable wound healing properties [15,16,17,18]. It is also weakly immunogenic, anti-inflammatory, anti-fibrotic, and releases bioactive factors that regulate cell growth and differentiation. However, HAM lacks cell-guiding ability to function optimally as an anisotropic cardiac patch. On the other hand, electrospun nanofiber can be easily fabricated into fibrous layers that resemble the architecture of cardiac natural ECM. Hence, by combining HAM and electrospun nanofibers, a mechanically competent cardiac patch that is topographically favorable can be produced for the regeneration of highly organized cardiac tissues.

Electrospun fiber-coated human amniotic membrane (EF-HAM) is an innovative composite scaffold design that has been increasingly studied for tissue engineering applications in recent years. Researchers have been studying the clinical utility of EF-HAM in the regeneration of urinary bladder [19], skin [20,21,22], cornea [23,24], and aligned tissue [14]. In previous studies, a fiber-coated HAM scaffold seeded with mouse adipose tissue-derived MSCs [22], and another with rabbit tenocytes and fibroblasts [25], have been shown to upregulate the expression of VEGF, bFGF, and PDGF in vitro. A silk fibroin-coated HAM scaffold seeded with adipose tissue-derived MSCs has also been shown to increase tissue vascularization and angiogenic factor expression when implanted in a mouse model of burn wounds [26]. However, to our knowledge, the influence of EF-HAM on the secretion of angiogenic factors from SkM has not yet been investigated.

In this study, we demonstrated the biocompatibility and ability of EF-HAM scaffolds to significantly induce the secretion of proangiogenic factors in SkM compared to the plain 2D culture. Multiplex factor analysis showed that potent angiogenic factors were detected at significantly elevated levels in CM generated from the SkM-seeded EF-HAM 7 min scaffolds. In vitro functional assays also demonstrated consistent findings, suggesting the angiogenicity of the EF-HAM 7 min scaffolds, possibly due to the highly secreted proangiogenic factors including angiopoietin-1 (Ang1), IL-8, and VEGF-C. CM obtained from SkM-seeded EF-HAM 7 min scaffolds was shown to promote greater cell viability, higher migration capacity, and longer capillary-like formation in HUVECs. Interestingly, it has been shown that the delivery of Ang1, IL-8 and VEGF-C could exert therapeutic benefits in the ischemic heart by promoting angiogenesis, maintaining vascular integrity, and inhibiting myocardial apoptosis [27,28,29].

The essential roles of Ang1 in the formation and maturation of blood vessels, as well as in the regulation of endothelial cell survival, have been previously reported [30,31]. Meanwhile, IL-8 has been demonstrated to directly enhance endothelial cell proliferation and survival, degradation of the extracellular matrix, cell migration and capillary tube formation [32]. Although VEGF-C plays a predominant role in lymphangiogenesis, it can also activate angiogenesis signaling pathways by binding to VEGFR-2. VEGF-C is as potent as VEGF-A in mediating the proliferative effect on endothelial cells and can stimulate a higher chemotactic response than VEGF-A at a low concentration. In addition, VEGF-C can induce the formation of dense capillaries with longer vessels [33]. In contrast, while angiogenin was highly secreted in HAM CM, HUVECs treated with HAM CM did not exhibit greater angiogenic activity than those treated with plain CM. This is probably due to the fact that angiogenin is merely a permissive factor required for other essential angiogenic factors to induce angiogenesis [34].

Recent studies have reiterated the role of scaffold topography in amplifying the angiogenic potential of stem cells via paracrine secretion. Fiber alignment has been postulated to potentiate the secretion of proangiogenic factors, including IL-8 and FGF-2, in bone marrow-derived MSCs [35]. In contrast, Ni Su et al., (2017) has demonstrated that mesh-like fibers with more complex orientations than random and aligned fibers facilitated greater production of proangiogenic factors, including VEGF and HGF, from adipose-derived MSCs [36]. Considering that we used composite scaffolds in this study, we believe that fiber topography alone may not be the sole governing factor responsible for mediating the secretion of angiogenic factors from SkM. The elevated angiogenic activity of our EF-HAM-induced CM might be the result of the unique interplay between the physical, mechanical and biological cues from both the aligned electrospun PLGA fibers and amniotic membrane. In future studies, the inclusion of PLGA fiber-only scaffolds can help us better evaluate the role of fiber topography in regulating the paracrine secretion of angiogenic factors.

It is important to note that the myoblast-fibroblast population ratio of the SkM might also be influenced by the physical and mechanical properties of the scaffolds resulting in the differential paracrine effects. Compared to the other scaffolds, the EF-HAM 7 min scaffold had thicker PLGA fibers, higher surface roughness and lower wettability. It is possible that these physical characteristics might favor the significantly increased proportion of skeletal fibroblasts on the EF-HAM 7 min scaffolds compared with those on the plain surface. Accordingly, the greater angiogenic potential of the EF-HAM 7 min scaffold could be attributed to increased proangiogenic factor secretion following the synergistic interaction between skeletal myoblasts and fibroblasts. This agrees with the results of Thummarati et al., (2020), who demonstrated that the co-culture of skeletal myoblasts with fibroblasts produced higher levels of VEGF and HGF and an increased angiogenic response in endothelial cells compared to the monoculture of either myoblasts or fibroblasts [37].

Taken together, our results suggest that the EF-HAM composite scaffold could potentiate the angiogenic paracrine activity of SkM and holds promising applications in the repair and regeneration of ischemic tissue. Further studies using scaffolds with more controlled topographical, physical and mechanical properties are required to elucidate their role in the stimulation of angiogenesis.

## 4. Materials and Methods

### 4.1. Skeletal Muscle Cells Isolation and Culture

The use of all human tissue samples in this study has obtained ethics approval from the Research Ethics Committee of The National University of Malaysia (Reference no.: UKM 1.5.3.5/244/02-01-02-SF1284 and UKM PPI/111/8/JEP-2017-411) with written consent from donors. Redundant skeletal muscle tissue was obtained aseptically from patients undergoing surgery from debridement or limb amputation at the National University of Malaysia (UKM) Medical Centre. Skeletal muscle cells (SkM) were isolated from processed tissue and cultured using protocols described in our previous study with some modifications [38]. Muscle tissue was minced and enzymatically digested with 0.25% trypsin-EDTA (TE; Sigma-Aldrich, St. Louis, MO, USA) to isolate SkM containing a mixture of skeletal myoblasts and fibroblasts. Isolated cells were cultured in Ham’s F10 medium (F10; Sigma-Aldrich) supplemented with 20% fetal bovine serum (FBS; Biowest, Nuaille, France) at 37 °C under humidified atmosphere with 5% CO_2_. Waste medium was replaced with fresh culture medium after cells had attached and subsequently changed every 48 h. From passage 1 onward, isolated SkM were grown on tissue culture surface coated with 10 ug/mL laminin (Sigma-Aldrich) for skeletal myoblast enrichment and cultured in an equal volume mix of Dulbecco’s modified Eagle’s medium—high glucose (DMEM; Sigma-Aldrich) and F10 medium supplemented with 10% FBS [39,40]. For experimental assays, SkM culture from passage 4–6 was used for cell seeding on tissue culture surfaces, HAM and EF-HAM scaffolds and maintained under the same culture condition as aforementioned unless stated otherwise. No laminin coating was applied to the surface of tissue culture plate or scaffold for any experimental assay.

### 4.2. HAM Processing and Decellularization

HAM was procured aseptically with consent from healthy mothers undergoing caesarian section at the UKM Medical Centre and processed within 24 h after retrieval as previously described [14]. Briefly, amnion was separated from chorion layer through blunt dissection, washed extensively in Dulbecco’s phosphate-buffered saline (DPBS; Thermo Fisher, Waltham, MA, USA), and soaked with moderate shaking in 0.05% sodium hypochlorite solution for 1 h to remove all blood residues and mucus. HAM was then washed multiple times for 3 × 15 min in DPBS and freeze-preserved in DPBS with 1% (*v*/*v*) antibiotic/antimycotic (AA; Thermo Fisher) solution at −80 °C until decellularization. To begin the decellularization process, smaller pieces of HAM (3 × 3 cm each) were incubated with 18.75 µg/mL of thermolysin (TL; Sigma-Aldrich) at 37 °C for 10 min, pulse vortexed for 60 times and washed multiple times in DPBS by moderate shaking to remove excess TL. Next, HAM pieces were briefly soaked in 0.25 M of sodium hydroxide (NaOH; BDH Laboratory Supplies, Leicestershire, UK) for 1 min, immediately pulse vortexed for 30 times and washed multiple times by moderate shaking in DPBS to remove excess NaOH. HAMs were later air-dried under biosafety cabinet and sent gamma sterilization. Sterilized, decellularized HAMs were stored under dry conditions at room temperature to maintain the shelf life until further use.

### 4.3. Fabrication of Composite EF-HAM Scaffolds

The fabrication of the composite EF-HAM scaffold was performed via electrospinning as previously described with some modifications [14,41]. Poly lactic-co-glycolic acid (50:50) (PLGA 50:50; DURECT, Birmingham, AL, USA) was dissolved at a 20% (*w*/*v*) concentration in a 7:3 solvent mixture containing dichloromethane (DCM; Sigma-Aldrich) and dimethylformamide (DMF; Sigma-Aldrich) and stirred at 230 rpm for 20 h at room temperature. PLGA was then spun onto the decellularized HAM attached to a rotating collector at 7.5 kV of applied voltage, 0.3 mL/h of polymer flow rate, and 15 cm of deposition distance. To study the effect of fiber thickness on the angiogenic potential of EF-HAM scaffolds, different fiber thicknesses were created on HAM by varying the deposition times to 3, 5, and 7 min. Resulting EF-HAM scaffolds were later air-dried under biosafety cabinet and UV-irradiated for 40 min.

The gross morphology and density of PLGA fibers on EF-HAM scaffolds were then observed through images captured using Olympus DSX1000 digital microscope. Prior to any cell culture application, HAM and EF-HAM scaffolds were rehydrated overnight in DPBS with 1% (*v*/*v*) AA solution.

### 4.4. Surface Roughness Measurement

The surface roughness of HAM and EF-HAM scaffolds was measured via optical method using a 3D Laser Confocal Microscope (Olympus LEXT OLS5000). The topological images of the scaffolds were acquired by the Olympus LEXT OLS5000 microscope, which was equipped with a 100× magnification objective lens (0.8 numerical aperture, 3.4 mm working distance and working field of view of 0.13 mm by 0.13 mm).

### 4.5. Scaffold Wettability

The surface contact angle of HAM and EF-HAM scaffolds was measured to determine scaffold wettability; 5 µL of distilled water was dispensed onto the surface of the scaffold by sessile drop method and its image captured 5 s after the water droplet has formed on the surface. The image of the water droplet was then analyzed using ImageJ to measure the water contact angle of the scaffold. Contact angle was determined from an average of at least five different measurements obtained from multiple spots on the scaffold.

### 4.6. Scanning Electron Microscopy

Surface ultrastructure and adhesion of SkM on HAM and EF-HAM scaffolds were observed on Zeiss Supra 55VP Field Emission Scanning Electron Microscope (FESEM). Scaffolds seeded with 10,000 cells/cm^2^ were grown for 48 h and fixed in 2.5% glutaraldehyde (Ajax Finechem, Wollongong, NSW, Australia) at 4 °C overnight in the dark. Samples were then rinsed for 3 × 5 min in phosphate buffered saline (PBS; Thermo Fisher), followed by rinsing for 2 × 10 min in deionized Milli-Q water. A series of ethanol (EtOH) dehydration steps was performed by soaking the samples in 50% (1 × 15 min), 75% (1 × 15 min), 95% (2 × 15 min) and 100% EtOH (3 × 20 min). Subsequently, samples were immersed in hexamethyldisilazane (HMDS; Nacalai Tesque, Kyoto, Japan) for 10 min, removed and stored to dry in a vacuum desiccator at room temperature. Samples were sputter-coated with gold at 30 mA for 30 s on specimen stubs before visualization under FESEM at 15 kV of acceleration voltage.

### 4.7. Live and Dead Assay

The viability of SkM on HAM and EF-HAM scaffolds was assessed through Live/Dead assay which distinguishes live from dead cells through staining with Calcein AM (Cayman Chemical, Ann Arbor, MI, USA) and ethidium homodimer-1 (EthD-1; Thermo Fisher), respectively. SkM were seeded on scaffolds at a 10,000 cells/cm^2^ seeding density and cultured for 48 h before staining procedure. For staining, cells were incubated with a mix solution of 2 µM of Calcein AM and 4 µM of EthD-1 prepared in Hank’s 1X Balanced Salt Solutions (HBSS; Cytiva, Marlborough, MA, USA) at room temperature for 30 min. Staining solution was then replaced with HBSS and the images of stained cells were captured at five different fields using fluorescence microscope (Nikon, Tokyo, Japan) before the number of live and dead cells were counted and averaged.

### 4.8. Conditioned Media Collection and Angiogenic Paracrine Factors Quantification

Myoblast-enriched SkM were seeded on the plain surface, HAM or EF-HAM scaffolds at 20,000 cells/cm^2^ of seeding density and grown for 5 days before replacing the culture medium with serum-free F10:DMEM loaded at 300 uL/cm^2^ of scaffold. After 72 h, the conditioned media (CM) were collected and centrifuged at 5000 rpm for 5 min to remove the cell debris [42]. CM were stored at −80 °C until further use to prevent protein degradation. The following Table 1 lists different groups of CM being collected and their respective culture conditions.

Using Magnetic Luminex Assay Kit (R&D Systems, Minneapolis, MN, USA), the concentration of multiple human angiogenic factors within the CM were quantified as per manufacturer’s protocols. In brief, magnetic microparticles pre-coated with antibodies for angiogenin, angiopoietin-1, angiopoietin-2, IL-8, bFGF, leptin, PlGF, VEGF-A and VEGF-C biomarkers were mixed with the CM to bind the analytes of interest. Following multiple washing steps and the addition of analyte-specific biotinylated antibody cocktail, streptavidin-phycoerythrin (PE) conjugate was added to the samples to bind to the biotinylated antibodies. The amount of analyte bound on the magnetic microparticles was measured as Median Fluorescence Intensity (MFI) using Luminex MAGPIX Analyzer (Manufacturer, country). The baseline fluorescence intensity of each factor in the non-CM control group was deducted from all MFI values in other CM groups. The corrected MFI values in each group were then normalized to Plain CM and presented as fold change secretion.

Following CM collection, SkM on the plain surface as well as HAM and EF-HAM scaffolds were fixed with 4% paraformaldehyde and the skeletal myoblasts were immunostained with mouse monoclonal anti-human desmin antibody (1:300 dilution in 1% goat serum) in order to distinguish them from skeletal fibroblasts as described previously [14]. SkM-loaded samples were then incubated with goat anti-mouse Alexa Fluor 488 secondary antibody (1:300 dilution) and counterstained with DAPI (1:15,000 dilution) to allow for the assessment of skeletal myoblasts and fibroblasts proportion on each scaffold.

### 4.9. HUVEC Viability, Migration and Tube Formation Assays

The angiogenic properties of CM were further evaluated by subjecting the CM to endothelial cell viability, migration and tube formation assays. For endothelial cell viability assay, HUVECs (Lonza, Basel, Switzerland) were seeded into a 96-well plate at a seeding density of 6000 cells/well and cultured in Endothelial Cell Growth Medium (PromoCell, Heidelberg, Germany) supplemented with 33% (*v*/*v*) CM at 37 °C and 5% CO_2_. At Day 2 and Day 5 of culture, the cell viability was assessed through incubation with 10% PrestoBlue Cell Viability reagent (Invitrogen, Waltham, MA, USA) for 4 h at 37 °C and 5% CO_2_ in the dark. The cell culture supernatant from each well was transferred into another 96-well plate and quantified for absorbance readings at 570 nm, which were later normalized to 600 nm reference wavelength.

For HUVEC migration assay, we conducted the assay using Falcon^®^ Permeable Support with 8.0 µm PET membrane (Corning, New York, NY, USA) in the presence of CM as chemoattractant. HUVEC growth medium containing only 2% fetal calf serum (FCS) was supplemented with 33% (*v*/*v*) CM and added into each well of the Falcon^®^ Companion 24-well plate. The cell culture insert was then aseptically placed into each well. Subconfluent HUVECs were harvested using Gibco™ TrypLE™ Select Enzyme (1×) (Thermo Fisher) and resuspended in serum-free HUVEC basal medium at a seeding density of 5 × 10^5^ cells/mL. Each of the inserts was applied with 100 µL of HUVECs and cell migration toward the bottom of the insert was allowed to happen during the incubation of the well plate at 37 °C and 5% CO_2_ for 5 h. Non-migrated cells that remained inside the insert were gently removed using cotton swabs. Migrated cells at the bottom of the insert were then fixed with 4% paraformaldehyde (Sigma-Aldrich) and washed three times in PBS. The cell culture inserts were then incubated in the dark with 4′,6-diamidino-2-phenylindole (DAPI; Thermo Fisher) at 1:15,000 dilution for 40 min at room temperature and were washed three times in PBS. Images of DAPI-stained nuclei at the bottom of the permeable membrane were captured at five different fields using fluorescence microscope (Nikon, Japan) before the number of nuclei were averaged.

We also performed an endothelial cell tube formation assay in the presence of CM using µ-Plate Angiogenesis 96 Well (Ibidi GmbH, Grafelfing, Germany) coated with Matrigel (Corning). Prior to plate coating, Matrigel was thawed and both the plate and pipette tips were chilled at 4 °C overnight. The Matrigel and plate were placed on ice to keep cold throughout the coating process. The inner wells of the plate were filled with 10 µL liquid Matrigel and incubated at 37 °C for 1 h to let the gel polymerized. Subconfluent HUVECs were trypsinized with 0.05% TE and resuspended at a seeding density of 4 × 10^5^ cells/mL. The upper wells of the plate were applied with 35 µL of HUVECs and later supplemented with 35 µL of CM. The plate was incubated at 37 °C and 5% CO_2_ in a humidified chamber and the formation of capillary-like structure on the Matrigel was captured using a live cell imaging system (NIS-Elements AR, Nikon, Tokyo, Japan) at 10× magnification at a 20-min interval for 12 h. Each experimental condition was run in triplicate wells and three representative image fields were captured from each well. Total tube length and number of loops from each image were quantified using Wimasis Image Analysis tool (Onimagin Technologies, Cordoba, Spain) before the measurements were averaged for each condition. A single tube was defined as a structure delimited by two junctions, while a single loop was formed when multiple tubes were connected end-to-end to form a closed circular structure.

### 4.10. Statistical Analysis

All assays were carried out using at least three independent biological samples and triplicates of each sample. All values were represented as mean ± SEM, where statistical significance was set at *p* < 0.05. Statistical analysis of means between experimental groups was performed by analysis of variance (ANOVA) using GraphPad Prism version 7.00 for Windows (GraphPad Software, San Diego, CA, USA).

## Figures and Tables

**Table 1:** Types of Conditioned Media Collected for Use in Angiogenesis Functional Assays.

Conditioned Medium	Culture Condition
Non-CM	Negative control; SkM-free, serum-free culture medium
Plain CM	Derived from SkM cultured on the plain tissue culture surface
HAM CM	Derived from SkM-seeded HAM scaffolds
EF-HAM 3 min CM	Derived from SkM-seeded EF-HAM 3 min scaffolds
EF-HAM 5 min CM	Derived from SkM-seeded EF-HAM 5 min scaffolds
EF-HAM 7 min CM	Derived from SkM-seeded EF-HAM 7 min scaffolds

## Data Availability

Not applicable.

## Abbreviations

ANOVA	Analysis of variance
CM	Conditioned media
DAPI	4′,6-diamidino-2-phenylindole
DPBS	Dulbecco’s phosphate-buffered saline
ECM	Extracellular matrix
EF	Electrospun fiber
EF-HAM	Electrospun fiber-coated human amniotic membrane
EtOH	Ethanol
FBS	Fetal Bovine Serum
FCS	Fetal Calf Serum
FESEM	Field Emission Scanning Electron Microscope
HAM	Human amniotic membrane
HUVEC	Human umbilical vein endothelial cell
MFI	Median fluorescence intensity
MI	Myocardial infarction
PBS	Phosphate buffered saline
PLGA	Poly lactic-co-glycolic acid
SkM	Skeletal muscle cells
TE	Trypsin-EDTA
